# Mortality Trend in Patients With Heart Failure and Psychoactive Substance Abuse in the United States—Pre‐ and Post‐COVID‐19 Pandemic Perspective (1999−2023)

**DOI:** 10.1002/clc.70425

**Published:** 2026-07-27

**Authors:** Emad Uddin Sajid, Ayesha Irfan, Muhammad Kashan, Syed Rayyan Ahmed, Muhammad Hasnain Azeem, Tooba Ali, Mabel Waqar, Muneeba Khan, Erum Shahzadi Malik, Md Ariful Haque

**Affiliations:** ^1^ Department of Medicine Dow University of Health Sciences Karachi Pakistan; ^2^ Dow Medical College DUHS Karachi Pakistan; ^3^ Dow University of Health Sciences Karachi Pakistan; ^4^ Dow Institute of Cardiology DUHS Karachi Pakistan; ^5^ Voice of Doctors Research School Dhaka Bangladesh; ^6^ Department of Public Health Atish Dipankar University of Science and Technology Dhaka Bangladesh

## Abstract

**Background:**

This study evaluates national trends in heart failure (HF) and psychoactive substance abuse‐related mortality among adults aged 25 years and older in the United States (US) from 1999 to 2023, stratified by sex, age, race/ethnicity, geographic regions, and the COVID‐19 pandemic period.

**Methods:**

Data from 1999 to 2023 were extracted from the CDC WONDER database. Age‐adjusted mortality rates (AAMR) and annual percentage changes (APC) were calculated using Joinpoint regression. ICD‐10 codes I50, I50.1, I50.9 (Heart Failure), and F10‐19 (Psychoactive Substance Use) were used to identify the conditions.

**Results:**

A total of 771 739 deaths were recorded from 1999 to 2023 among individuals aged 25 and older. Most deaths occurred in medical facilities (39.24%), followed by decedents' homes (34.14%), long‐term care facilities (16.30%), hospice (6.65%), and other settings (3.67%). The AAMR increased markedly from 1.68 in 1999 to 20.7 in 2023 (AAPC of 12.09, 95% confidence interval [CI]: 10.97−14.26). A substantial increase was observed from 1999 to 2005 (APC: 38.5%, 95% CI: 29.1–55.8), followed by a slower increase until 2020 (APC: 5.68%, 95% CI: 5.02–14.8). Men showed consistently higher AAMRs than females (18.8 vs. 8.65). By age group, mortality was highest among individuals aged 65 years and older.

**Conclusion:**

Using CDC WONDER data (1999–2023), we found a sharp rise in HF mortality linked to psychoactive substance abuse, from 1.68 to 20.7 per 100 000. Mortality burden was highest among males, older adults, non‐Hispanic White individuals, and rural populations, highlighting widening demographic and geographic disparities.

## Graphical abstract

From 1999 to 2023, national HF‐substance abuse‐related mortality rates in adults rose over 10‐fold, disproportionately affecting males, older adults, and NH White and NH Black populations. Geographically, nonmetropolitan areas and the Midwestern/Northwestern regions saw the highest burden, signaling an urgent need for integrated cardiovascular and addiction care.

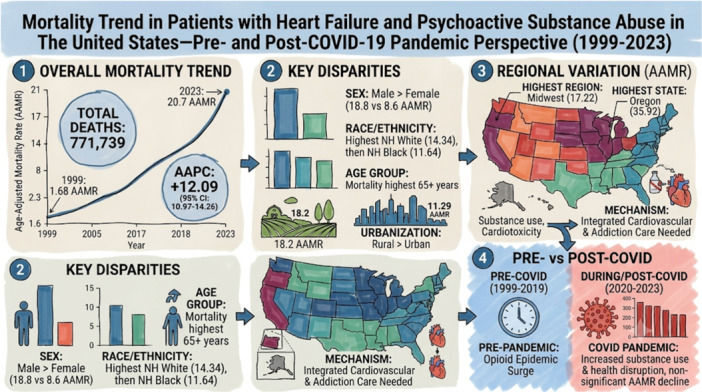

## 1 Introduction

Heart failure (HF) is a clinical syndrome characterized by the inability of the heart to pump effectively due to structural or functional abnormalities affecting ventricular contraction or filling [1]. HF is one of the most common cardiovascular disorders worldwide, with a high morbidity and mortality. Currently, nearly 6.5 million Americans are living with HF [2]. It imposes a significant burden on the healthcare system, contributing to increased hospitalizations and frequent readmissions.

The use of psychoactive substances, including alcohol, cocaine, methamphetamine, opioids, cannabis, and synthetic drugs, has increased, particularly in the younger population [3]. It can directly or indirectly impact cardiovascular health [4]. Psychoactive substances can cause myocardial toxicity, arrhythmia, hypertensive crisis, and cardiomyopathy (CMP), all of which may lead to HF [5]. Chronic use of these substances has been associated with less favorable HF outcomes because of poor lifestyle, nonadherence to medications, and other comorbid psychiatric illnesses. In the past decade, over 10 million people have been hospitalized with a primary diagnosis of HF. Among them, substance abuse was among the most frequently co‐documented conditions and has been associated with particularly poor outcomes in prior studies [4].

From 1999 to 2023, the mortality rate due to HF and psychoactive substance abuse has steadily increased in the United States. While the majority of deaths are seen in older adults aged more than 65 years, in the younger age group, the mortality rate has also risen over the past two decades. Patients with both HF and psychoactive substance abuse are mostly older and have a lower proportion of females compared to males [4].

This study aims to evaluate and characterize the trends in mortality co‐documented with both HF and psychoactive substance abuse among individuals aged 25 years or older in the United States from 1999 to 2023. This study specifically aims to identify and explain the demographic disparities, stratified by sex, race, age, geographical regions, and the COVID‐19 pandemic period, thus offering a comprehensive epidemiological basis to guide focused interventions, policy decisions, and clinical management policies.

## 2 Methods

### 2.1 Study Design

The data used in this study were sourced from the Centers for Disease Control and Prevention's Wide‐ranging Online Data for Epidemiologic Research (CDC‐WONDER) database, a widely used and well‐established database of death records [6]. This database is accessible to the public and healthcare professionals [7]. Given the public nature of this data, International Review Board (IRB) approval was not required. This study strictly adhered to the Strengthening the Reporting of Observational Studies in Epidemiology (STROBE) guidelines [8].

### 2.2 Study Population

This study included adults aged 25 years or older and mortality from HF and psychoactive substance abuse from 1999 to 2023 in the United States. We utilized data from the Multiple Causes of Death Public Use Registry to identify deaths due to HF among people with substance abuse. The International Classification of Diseases (ICD) codes included were I50 for HF and F10 to F19 for psychoactive substance use. Specifically, both ICD‐10 code I50 (Heart Failure) and codes F10–F19 (Psychoactive Substance Use) were required to appear concurrently as contributing causes on the same death certificate, with neither restricted to the underlying cause of death (UCOD). Both conditions were therefore identified from the multiple‐cause‐of‐death (MCOD) fields rather than the UCOD field alone.

### 2.3 Data Extraction

The data elements extracted included age from 25 to 85+ years, race/ethnicity (Hispanics, NH White, NH Black or African American, NH Asian, and American Indians), US census region (Northeast, Midwest, South, and West), urban−rural classification, and place of death (medical facility, hospice facility, decedent's home, nursing home, and others). The population was categorized according to the National Center of Health Statistics Urban−Rural classification scheme, classifying counties as urban (including large central metropolitan, large fringe metropolitan, medium metropolitan, and small metropolitan) or rural (comprising micropolitan and noncore areas) based on the 2013 US Census classifications [9]. Analyses of urban−rural trends were confined to the years 1999–2020, as post‐2020, the CDC WONDER database shifted from a binary urban–rural classification to a more nuanced multi‐level urbanization paradigm, hence restricting direct comparability with prior years. State‐level analyses were conducted for two distinct periods: 1999–2020 using bridged‐race estimates (Supporting Information S1: Table 7) and 2021−2023 using single‐race estimates from the expanded MCD file (Supporting Information S1: Table 11), with the latter presented for descriptive reference only. We also stratified our data by the COVID‐19 pandemic, as substance abuse is significantly associated with COVID‐19 outcomes [10]. All psychoactive substance categories encompassed by codes F10–F19, including alcohol, opioids, cannabis, cocaine, stimulants, and other psychoactive substances, were analyzed as a composite group rather than disaggregated by individual substance type.

### 2.4 Statistical Analysis

Crude and age‐adjusted mortality rates (AAMR) per 100 000 population were computed using the CDC WONDER database to characterize national mortality patterns, stratified by sex, age, race, and geographic location to examine disparities. Temporal trends were evaluated using the Joinpoint Regression Program (Joinpoint V 4.9.0.0, National Cancer Institute, Bethesda, MD, USA) to estimate the annual percent change (APC) and average annual percent change (AAPC), with statistical significance set at *p* < 0.05.

## 3 Results

### 3.1 Overall

**Figure 1:**
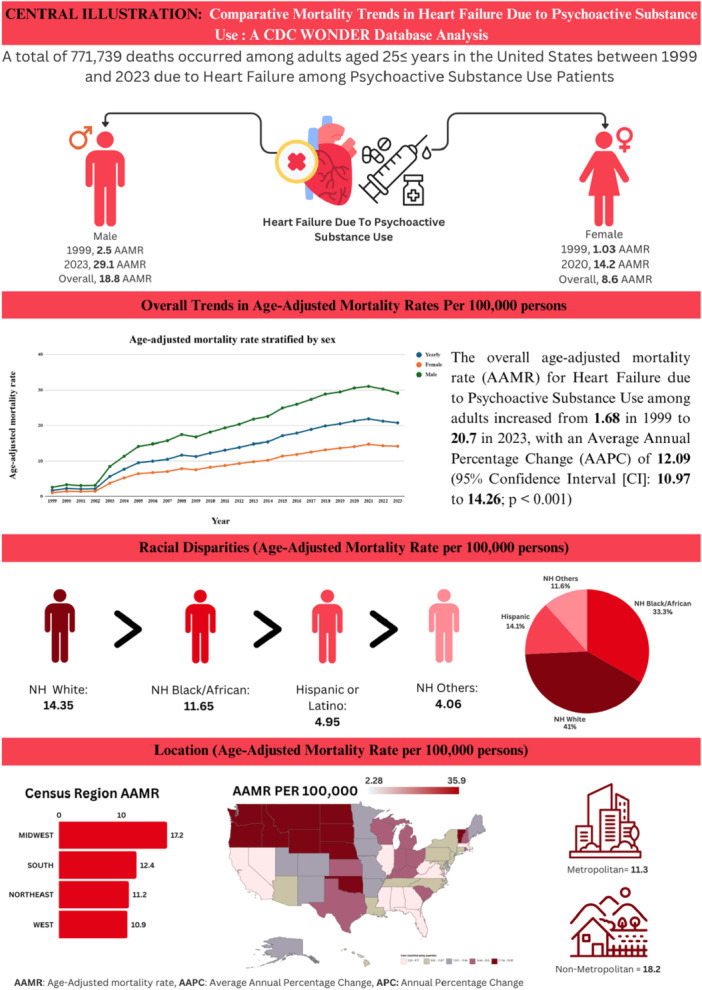
Central image for patients with heart failure and psychoactive substance abuse.

**Figure 2:**
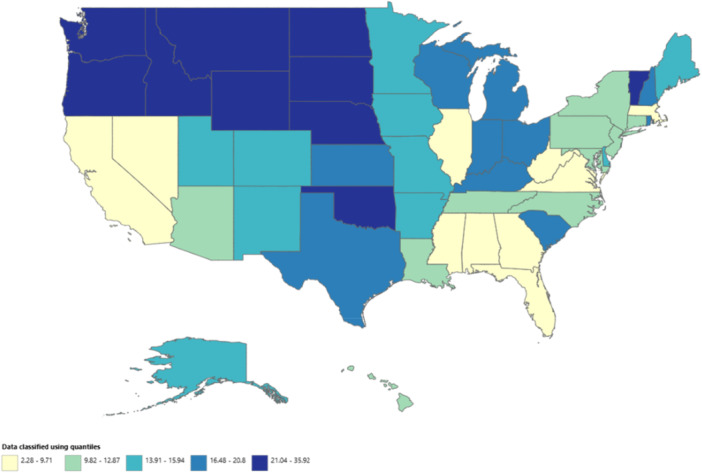
A map displays the differences in age‐adjusted mortality rates among US states for patients with heart failure and psychoactive substance abuse.

**Figure 3:**
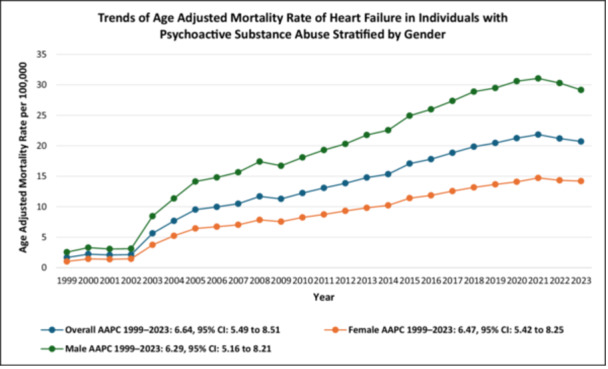
Temporal trends of age‐adjusted mortality rate in patients with heart failure and psychoactive substance abuse stratified by sex.

Between 1999 and 2023, a total of 771 739 deaths related to HF and psychoactive substance abuse occurred among individuals aged 25 years and above in the United States (Supporting Information S1: Tables 1 and 3). These fatalities were distributed across several settings, with the largest proportion occurring in medical facilities (39.24%), followed by deaths at decedents' homes (34.14%), nursing homes or long‐term care facilities (16.30%), hospice facilities (6.65%), and other locations (3.67%) (Supporting Information S1: Table 2) (Figure 1). Figure 2 provides a comprehensive illustration summarizing the characteristics and findings of the study. Figure 3 illustrates state‐level variation in AAMR in patients with HF caused by psychoactive substance abuse in the United States.

### 3.2 Annual Trends

The AAMR for deaths due to HF and psychoactive substance abuse exhibited a significant increase, rising from 1.68 per 100 000 people in 1999 to 20.71 in 2023. The AAPC was 12.09 (95% confidence interval [CI]: 10.97−14.26), with a *p* < 0.01. Notably, a sharp rise in the AAMR occurred between 1999 and 2005 (APC: 38.53, 95% CI: 29.12−55.86, *p* = 0.002), followed by a steady increase from 2005 to 2020 (APC: 5.68, 95% CI: 5.02−14.82, *p* < 0.01). Of note, raw death counts increased nearly threefold between 2002 and 2003 (from 3978 to 10 569), contributing substantially to the steep APC observed during the 1999–2005 segment. Between 2020 and 2023, however, there was a slight decline in AAMR (APC: −1.53, 95% CI: −11.01 to 4.41, *p* = 0.53) (Supporting Information S1: Table 10).

### 3.3 Stratified by Sex

From 1999 to 2002, males had a slightly higher mortality rate than females. However, starting in 2003, this gap widened, with males exhibiting substantially higher AAMRs than females. The overall AAMR for males was 18.81 (95% CI: 18.54−19.08), while for females, it was 8.65 (95% CI: 8.50−8.80). Both male and female mortality rates increased from 1999 to 2023 (males: AAPC: 11.79, 95% CI: 10.73−13.76, *p* < 0.01; females: AAPC: 12.11, 95% CI: 11.18−13.91, *p* < 0.01).

**Figure 4:**
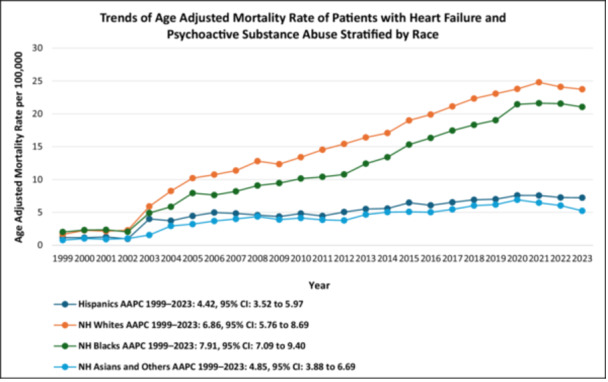
Temporal trends of age‐adjusted mortality rate in patients with heart failure and psychoactive substance abuse stratified by race.

Analysis stratified by sex revealed similar trends in age‐adjusted mortality for HF and psychoactive substance abuse across both genders, with some variations in magnitude and specific APC. AAMRs for both males and females increased sharply between 1999 and 2005 [males: APC: 38.37, 95% CI: 29.31−53.21, *p* < 0.01; females: APC: 38.25, 95% CI: 29.58−50.04, *p* < 0.01], followed by a steady increase from 2005 to 2020 [males: AAPC: 5.45, 95% CI: 4.80−7.14, *p* < 0.01; females: APC: 5.58, 95% CI: 5.04−14.02, *p* < 0.01]. From 2020 to 2023, AAMRs declined slightly for both males and females [males: APC: −2.29, 95% CI: −11.6 to 2.56, *p* = 0.29; females: APC: −0.51, 95% CI: −8.35 to 4.54, *p* = 0.85] (Supporting Information S1: Table 5 and Figure 4).

### 3.4 Stratified by Age

Significant differences in mortality rates were observed across age groups. The highest number of deaths occurred among individuals aged 65 years and older (631 274), followed by individuals aged 46−65 years (130 319), and the lowest mortality was seen in individuals aged 25−45 years (10 146). The AAMRs followed a similar pattern, with the highest rates observed in the 65+ years age group (54.72 [95% CI: 54.06−55.38]), followed by the 46−65 years age group (5.78 [95% CI: 5.63−5.94]) and the 25−45 years age group (0.50 [95% CI: 0.45−0.55]).

**Figure 5:**
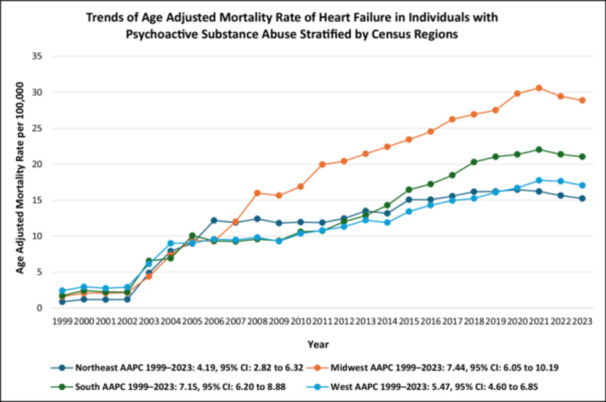
Temporal trends of age‐adjusted mortality rate in patients with heart failure and psychoactive substance abuse stratified by census region.

The AAMRs for all age groups showed significant increases between 1999 and 2023, with the greatest relative rise observed in the 65+ years age group (AAPC: 11.08, 95% CI: 10.11−13.51, *p* < 0.01), followed by the 46−65 years group (AAPC: 10.21, 95% CI: 9.60−11.36, *p* < 0.01) and the 25−45 years group (AAPC: 9.27, 95% CI: 8.43−10.85, *p* < 0.01) (Supporting Information S1: Table 4 and Figure 5).

### 3.5 Stratified by Race

There was considerable variation in the number of deaths among different racial and ethnic groups. The highest number of deaths occurred among NH White individuals (663 831; 86.01%), followed by NH Black or African American individuals (68 788; 8.91%), Hispanic individuals (24 175; 3.13%), and NH Asian or other groups (American Indian or Alaska Native and Asian or Pacific Islanders) (11 911; 1.54%). AAMRs were highest among NH White individuals, followed by NH Black or African American, Hispanic or Latino, and NH Asian or other groups. The overall AAMR for NH White individuals was 14.34 (95% CI: 14.17−14.51), for NH Black or African Americans was 11.64 (95% CI: 11.20−12.08), for Hispanic or Latino was 4.94 (95% CI: 4.61−5.28), and for NH Asians and others was 4.05 (95% CI: 3.67−4.45).

NH Whites exhibited a dramatic 10‐fold rise in AAMRs from 1999 to 2005 (APC: 38.88, 95% CI: 30.55−49.59, *p* < 0.01), followed by a nearly twofold increase from 2005 to 2020 (APC: 5.98, 95% CI: 5.40−7.48, *p* < 0.01). From 2020 onwards, AAMRs slightly declined (APC: −1.11, 95% CI: −9.50 to 3.48, *p* = 0.60).

**Figure 6:**
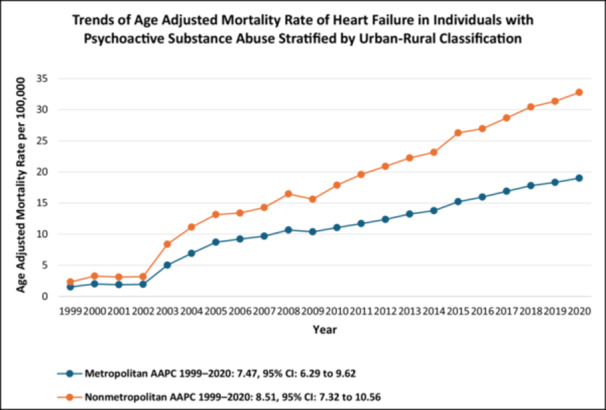
Temporal trends of age‐adjusted mortality rate in patients with heart failure and psychoactive substance abuse stratified by urbanization.

The AAMRs for NH Black or African American, Hispanic, and NH Asian and Other (American Indian or Alaska Native and Asian or Pacific Islander) individuals showed steady increases from 1999 to 2023, with the rise being most prominent among NH Black or African American individuals [AAPC: 11.18, 95% CI: 10.30−13.01, *p* < 0.01], followed by Hispanic individuals [AAPC: 9.56, 95% CI: 7.92−13.43, *p* < 0.01] and NH Asians and others [AAPC: 9.35, 95% CI: 7.99−12.01, *p* < 0.01] (Supporting Information S1: Table 6 and Figure 6).

### 3.6 Stratified by Geographical Regions

Significant geographical variation in AAMRs was noted across different states for the 1999−2020 period. AAMRs ranged from as low as 2.28 (95% CI: 2.24−2.32) in California to as high as 35.92 (95% CI: 35.45−36.39) in Oregon. States in the top 90th percentile for AAMR included Oregon, North Dakota, Montana, Vermont, Washington, Wyoming, and Idaho, which exhibited AAMRs approximately four times higher than those in states in the lower 10th percentile, including California, Alabama, the District of Columbia, Florida, Georgia, Massachusetts, Mississippi, Virginia, and West Virginia (Supporting Information S1: Table 7).

For the 2021–2023 period, using single‐race population estimates (Supporting Information S1: Table 11), AAMRs ranged from as low as 3.76 (95% CI: 3.63–3.89) in California to as high as 53.64 (95% CI: 52.25–55.03) in Oregon. States in the top 90th percentile for AAMR included Oregon, Kentucky, Washington, Wyoming, Minnesota, and Vermont, which exhibited AAMRs approximately four times higher than those in states in the lower 10th percentile, including California, the District of Columbia, Connecticut, New York, New Jersey, and Florida (Supporting Information S1: Table 11).

**Figure 7:**
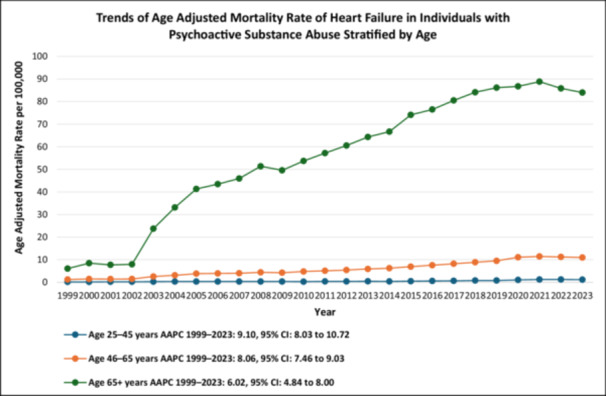
Temporal trends of age‐adjusted mortality rate in patients with heart failure and psychoactive substance abuse stratified by age.

Throughout the study period, the highest mortality was observed in the Midwestern region (AAMR: 17.22, 95% CI: 16.88−17.56), followed by the Southern region (AAMR: 12.38, 95% CI: 12.15−12.61), the Northeastern region (AAMR: 11.16, 95% CI: 10.86−11.46), and the Western region (AAMR: 10.92, 95% CI: 10.64−11.21) (Supporting Information S1: Table 8 and Figure 7).

### 3.7 Stratified by Urbanization

**Figure 8:**
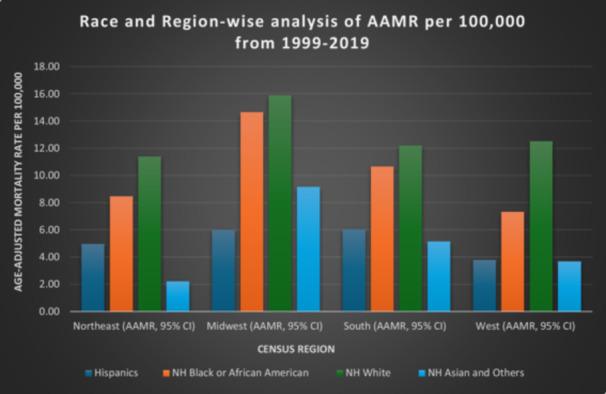
Race and region‐wise analysis of AAMR per 100 000 in heart failure and psychoactive substance abuse‐related mortality in adults from 1999 to 2019.

Rural areas consistently exhibited higher AAMRs than urban areas throughout the study period, with overall AAMRs of 18.20 (95% CI: 18.11–18.29) and 11.29 (95% CI: 11.25–11.32), respectively. AAMRs increased significantly in both settings from 1999 to 2020, with a slightly greater increase observed in urban areas (AAPC: 13.80, 95% CI: 12.81–15.83, *p* < 0.01) than in rural areas (AAPC: 13.10, 95% CI: 11.97–15.99, *p* < 0.01). Despite the higher AAPC in urban areas, rural areas maintained higher AAMRs throughout the study period (Supporting Information S1: Table 9 and Figure 8).

### 3.8 Stratified by COVID‐19 Pandemic

For this stratification, the pre‐ and post‐pandemic periods were defined using a 2019/2020 cutoff to align with the World Health Organization's declaration of COVID‐19 as a pandemic in early 2020. This differs from the 2020/2021 cutoff used elsewhere in this study (e.g., state‐level and race‐stratified analyses), which reflects the CDC's transition from bridged‐race to single‐race population estimates rather than the pandemic itself (see Limitations).

#### 3.8.1 The Pre‐Pandemic Period (1999−2019)

**Figure 9:**
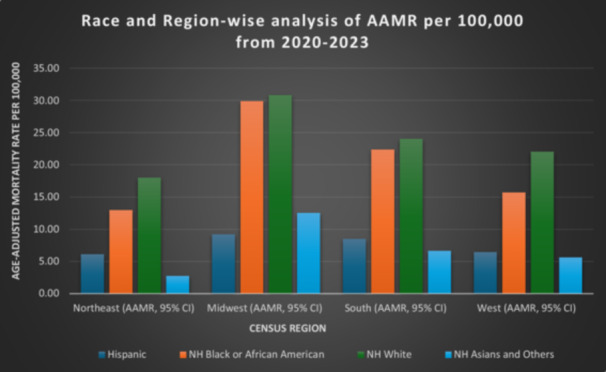
Race and region‐wise analysis of AAMR per 100 000 in heart failure and psychoactive substance abuse‐related mortality in adults from 2020 to 2023.

NH Black or African American populations had the highest mortality rate in the Midwest (14.64 per 100 000), followed by the South (10.65 per 100 000), Northeast (8.46 per 100 000), and West (7.32 per 100 000). The NH White populations had the highest mortality rate in the Midwest (15.89 per 100 000), followed by the South (12.20 per 100 000), West (12.51 per 100 000), and Northeast (11.38 per 100 000). Hispanics exhibited the highest mortality rate in the South (6.05 per 100 000), followed by the West (5.14 per 100 000), Midwest (5.98 per 100 000), and Northeast (4.97 per 100 000). NH Asian and Others (American Indian or Alaska Native and Asian or Pacific Islanders) had the highest mortality rate in the West (3.79 per 100 000), followed by the South (5.14 per 100 000), Midwest (9.15 per 100 000), and Northeast (2.21 per 100 000) (Figure 9).

#### 3.8.2 During and Post‐Pandemic (2020−2023)

**Figure 10:**
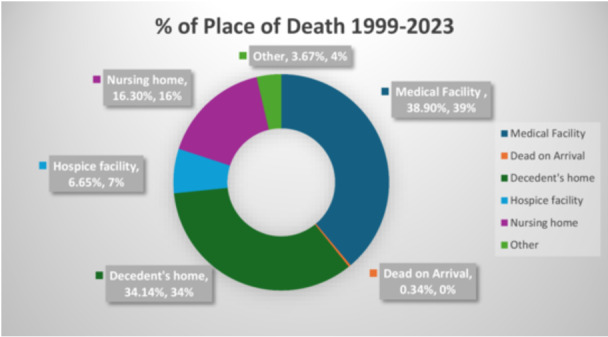
Percentage of place of death in heart failure and psychoactive substance abuse‐related mortality in adults from 1999 to 2023.

NH Black or African American populations had the highest mortality rate in the Midwest (29.89 per 100 000), followed by the South (22.37 per 100 000), Northeast (12.99 per 100 000), and West (15.73 per 100 000). NH White populations had the highest mortality rate in the Midwest (30.83 per 100 000), followed by the South (24.02 per 100 000), West (22.02 per 100 000), and Northeast (18.00 per 100 000). Hispanics showed the highest mortality rate in the Midwest (9.24 per 100 000), followed by the South (8.49 per 100 000), West (6.45 per 100 000), and Northeast (6.12 per 100 000). NH Asian and Others (American Indian or Alaska Native and Asian or Pacific Islanders) had the highest mortality rate in the Midwest (12.54 per 100 000), followed by the South (6.64 per 100 000), West (5.64 per 100 000), and Northeast (2.78 per 100 000) (Figure 10).

## 4 Discussion

The prevalence and potency of substance abuse use have increased over the past two decades, with the majority of deaths occurring in medical facilities, with the burden disproportionately affecting males, older adults, NH White and NH Black or African American populations, and nonmetropolitan regions, particularly in the Midwest and the Northwest. Prior studies have demonstrated a sharp nationwide rise in substance use–related cardiovascular mortality. One analysis found that age‐adjusted substance‐use‐plus‐CVD mortality rose from 9.9 to 21.4 per 100 000 between 1999 and 2019 (AAPC ≈ 4% per year) [11].

Alcohol was involved in 65% of these deaths, with opioids, cocaine, and stimulants contributing roughly 14%, 10%, and 6%, respectively [11]. Notably, amphetamines/stimulants showed the fastest increase (AAPC ~18% from 1999 to 2019) [11]. In parallel, national data on HF hospitalizations document a methamphetamine‐related HF epidemic: meth‐associated HF admissions grew from about 550 in 2002 to over 6600 by 2014 [12]. Collectively, these findings suggest that the rising use of cardiotoxic substances (methamphetamines, cocaine, opioids, and heavy alcohol) may be contributing to the overall increase in HF deaths. These findings are further supported by a recent analysis of self‐injury mortality among individuals with mental and behavioral disorders, which similarly documented a dramatic rise in substance use‐related mortality from 1999 to 2023 using CDC WONDER data, with substance use disorders accounting for approximately 90% of all self‐injury deaths during this period [13].

Prior experimental and clinical studies have shown that psychotropic drugs have extensive effects on the cardiovascular system. Methamphetamines cause tachycardia, hypertension, myocardial ischemia, and even aortic dissection [14, 15]. Chronic methamphetamine use may lead to CMP and HF [14]. Additional evidence indicates that chronic cocaine use may produce diffuse cardiac injury, provoking vasoconstriction, sodium/potassium channel blockade, and increased myocardial oxygen demand, which has been shown to precipitate myocardial infarction and CMP [16]. A recent meta‐analysis confirmed that long‐term cocaine users develop structural cardiac changes consistent with HF, especially diastolic dysfunction [17]. Although opioids are not classically cardiotoxic, they have important cardiovascular effects. Acute overdose can cause hypotension, bradycardia, and hypoxemia, and chronic use has been associated with arrhythmias or CMP [18, 19]. One hospitalization study found 8.6% of opioid overdose patients developed a cardiovascular event, and 0.7% had acute HF [20]. Consistent with alcohol's outsized contribution to substance‐related cardiovascular mortality noted above [21], chronic heavy alcohol consumption has been associated with dilated CMP and HF in prior studies, presenting like non‐ischemic HF [22].

The sharp increase in AAMR during 1999−2005 may correspond to the early stages of the opioid epidemic. In the late 1990s and early 2000s, there was a marked escalation in the prescription of opioid medications, particularly oxycodone and hydrocodone, driven in part by aggressive pharmaceutical marketing and the underestimation of addiction risk [23]. This rise in prescription opioid misuse has been accompanied by an increasing burden of cardiovascular complications, including arrhythmias, CMP, and HF [18, 19]. Following the initial surge, the continued but slower rise in mortality during 2005−2020 may reflect the transition from prescription opioid misuse to heroin and synthetic opioids such as fentanyl [24]. After 2013, synthetic opioids, particularly fentanyl, became increasingly implicated in opioid‐related deaths, reflecting their growing prevalence and potency, while also contributing to an increased burden of cardiovascular toxicity [24, 25]. By January to June 2018, illicitly manufactured fentanyl was involved in approximately two‐thirds of opioid deaths [25]. Moreover, increasing polysubstance use (e.g., opioids combined with stimulants like cocaine or methamphetamine) may have further amplified cardiotoxic effects and increased the risk of HF [26]. The slight decline in HF mortality after 2020 was not statistically significant but may reflect the effects of the COVID‐19 pandemic. Disruptions in healthcare access, changes in substance use patterns, and shifts in mortality reporting during the pandemic may have influenced observed HF mortality trends. Additionally, the almost threefold rise in raw death counts between 2002 and 2003 (from 3978 to 10 569) may be partially due to the staggered and inconsistent adoption of the revised 2003 US Standard Certificate of Death, that is, the first significant revision since 1989, which resulted in discrepancies in cause‐of‐death reporting among states during this transitional phase, and cannot be solely ascribed to a genuine epidemiological increase [27].

There was a striking rise in HF and psychoactive substance abuse deaths in both sexes, although males consistently experienced a substantially higher mortality burden throughout the study period. The widening gap in AAMRs between males and females after 2003 may reflect a combination of behavioral, biological, and systemic factors. One potential explanation is the higher prevalence and intensity of substance use among men. Studies have consistently shown that males are more likely to use high‐risk substances strongly associated with cardiotoxic effects [28]. Biological factors may also contribute to this disparity. Estrogen has been shown to exert cardioprotective effects, including reduction of oxidative stress, and thereby, attenuation of adverse cardiac remodeling, which may confer relative protection against substance‐induced myocardial injury in premenopausal females, potentially explaining the lower female mortality burden observed in our data [29]. Moreover, sex‐based disparities in substance‐specific cardiotoxicity have been established, especially with methamphetamine. Experimental models have indicated increased methamphetamine‐induced dilated CMP and worse survival in males compared to females, with similar observations reported in human patients [30]. Similarly, a large study found that among methamphetamine users, male sex was independently associated with an increased risk of myocardial infarction [31]. The widening male–female gap after 2003 may also coincide with the escalating prescription opioid epidemic and increasing stimulant use, which disproportionately affected men. This divergence may have widened further after 2010 as the epidemic shifted toward illicit substances such as heroin, methamphetamine, and synthetic opioids. During this phase, drug overdose mortality increased more rapidly among men than women, possibly reflecting men's greater tendency to engage in higher‐risk substance use behaviors, including polydrug use, consumption of larger quantities, use of more potent drugs, and obtaining drugs from unregulated sources [32]. Consistent with our findings, Ahmed et al. similarly reported substantially higher mortality rates among males compared with females across the 1999−2023 study period [13]. However, a recent CDC‐based study reported that substance use−associated cardiovascular disease mortality increased more rapidly among females than males between 1999 and 2019 [11]. A similar pattern was observed in our study: although males maintained higher absolute AAMRs throughout the study period, females exhibited a greater AAPC, indicating a steeper relative increase in mortality over time. However, because this study is descriptive and ecological in nature, the proposed mechanisms underlying these sex differences cannot be directly evaluated using the available data and should be explored in future studies.

There was a clear age‐stratified disparity in mortality rates due to HF among individuals with substance use disorders, with the highest AAMRs and absolute deaths observed in the 65+ years age group. This pattern is expected, given that aging is a primary risk factor for cardiovascular disease, including HF, due to cumulative exposure to comorbidities such as hypertension, diabetes, and atherosclerosis [33, 34]. In older adults, the physiologic reserve declines, and cardiac remodeling accelerates in response to both intrinsic aging processes and external insults like chronic substance use. Moreover, older individuals with substance use disorders may have prolonged exposure to cardiotoxic agents such as alcohol, which may exacerbate myocardial dysfunction over time. Absolute HF mortality in 25–44‐year‐olds remains low, but the rate is increasing substantially in this group (AAPC: 9.27%). Although the 65+ years age group demonstrated the greatest relative increase (AAPC: 11.08%), the substantial rise among younger adults is clinically significant and warrants attention. This pattern coincides with recent trends in the drug epidemic. Synthetic opioids (particularly fentanyl) and stimulants (notably methamphetamine) have surged in popularity among young users [35]. These high‐potency drugs have acute cardiotoxic effects. Case series increasingly describe methamphetamine‐associated CMP in young adults with few traditional risk factors. Compounding the problem, polysubstance use is very common in this age group: about 20% of young adults report using multiple drugs concurrently [35].

In our analysis, NH White individuals bore by far the greatest burden, accounting for 86.0% of the total deaths, followed by NH Black or African American individuals, who accounted for 8.91% of total mortalities. Hispanic and NH Asian/Other groups accounted for substantially smaller proportions of total deaths. NH White AAMR rose sharply during 1999–2005, continued increasing until 2020, then declined slightly, whereas NH Black or African American, Hispanic, and NH Asian/Other AAMRs rose steadily through 2023, with the fastest rise in NH Black or African Americans. These results mirror national data showing that roughly 70% of substance‐related cardiovascular deaths involve NH White Americans [11], while recent overdose trends have been most acute in NH Black or African American communities [36]. By contrast, Hispanic and NH Asian/Other populations have persistently lower AAMRs, consistent with their generally lower prevalence of heavy substance use and overdose mortality [11, 36]. National surveys show that NH White adults report higher rates of heavy alcohol use (≈7–8% of adults per month) than NH Black or African American, Hispanic, or NH Asian/Other adults [36], which may contribute to the NH White excess in HF mortality. The opioid epidemic initially affected rural, predominantly NH White geographical areas in the 2000s, amplifying HF from opioid‐induced CMP in those communities. In contrast, NH Black or African Americans have been disproportionately affected by rising stimulant and illicit opioid overdoses in the last decade [37]. For example, stimulant‐related deaths have increased most rapidly among NH Black or African American and Native American populations [38]. These evolving drug epidemics may partly be associated with the acceleration in NH Black or African American HF mortality rates; although their absolute AAMR remains lower than NH Whites, their relative increase has been the greatest. Certain racial and ethnic groups, particularly NH Black or African American and Hispanic individuals, carry a higher prevalence of hypertension, diabetes, obesity, and other cardiometabolic risk factors that predispose to HF [39]. It is plausible that these comorbidities could exacerbate the cardiac toxicity of substance use. Similarly, non‐Hispanic Black or African American individuals demonstrated the steepest rise in mortality rates in the recent contemporaneous analysis by Ahmed et al., mirroring the accelerating trend observed in our data [13].

Geographically, our analysis showed striking state‐level and regional differences in mortality, which reflect underlying patterns of drug use, socioeconomic conditions, and health infrastructure. For example, many of the highest‐AAMR states (Oregon, Wyoming, Idaho, Washington, Montana, etc.) are in the West and Northern Plains, regions where stimulant use, especially methamphetamine, has been reported to be prevalent. National hospital data indicate that methamphetamine‐associated HF cases are concentrated on the West Coast [12].

The urban−rural disparity in psychoactive substance abuse and HF mortality is also striking: nonmetropolitan areas had a far higher absolute mortality rate than metropolitan areas (overall AAMR: 18.20 vs. 11.29), consistent with recent comparative studies [40, 41] that have documented higher death rates and slower mortality improvements in rural communities. However, our analysis found that urban areas showed a marginally faster rate of increase (AAPC: 13.80 vs. 13.10), which diverges from broader substance use‐CVD trends that have documented more pronounced increases in nonmetropolitan areas and warrants further investigation [42]. Several interrelated factors underlie the persistently higher absolute mortality burden in rural areas. Rural communities face healthcare workforce shortages and facility closures; over 130 rural hospitals have shut down since 2010, reducing local capacity to manage cardiac complications [43]. A recent national study noted that rural residents have poor access to healthcare services for urgent conditions and tend to receive lower‐quality care [44], implying that substance‐related emergencies (including decompensated HF) are more likely to be fatal or delayed. Socioeconomic stress and stigma surrounding substance use are also more pronounced in rural communities, where higher poverty rates, limited employment opportunities, and stronger social stigma delay care‐seeking [45]. Prevention and treatment resources are also sparser. Medication‐assisted treatment (MAT) for opioid use, a life‐saving intervention, is far less available in rural areas [46]; for example, in 2011, approximately 60% of rural counties had an MAT shortage versus only 5% of metropolitan counties [47]. As a result, rural overdose and cardiotoxic drug use have increased substantially; a recent report found that drug deaths from psychostimulants were significantly higher in rural counties [48].

## 5 Limitations

This study has several limitations inherent to its retrospective, registry‐based design. First, the use of death certificate data from the CDC WONDER database may be subject to misclassification, bias, or underreporting of causes of death, particularly regarding psychoactive substance abuse and HF, which can often be influenced by comorbid conditions and clinician judgment. Furthermore, the nearly threefold rise in recorded deaths from 3978 in 2002 to 10 569 in 2003 may partially indicate a coding or reporting artifact due to changes in death certificate standards; thus, trend estimations during this period should be approached with caution.

Second, the reliance on ICD coding may not fully capture the nuance of substance use disorders, leading to potential underestimation or overestimation of mortality rates. Moreover, alterations in HF coding methodologies throughout the 24‐year research duration may have affected the identification of HF‐related fatalities, resulting in temporal discrepancies that cannot be entirely accounted for in a registry‐based study of this nature. Additionally, the use of ICD‐10 codes F10–F19 as a composite category precluded disaggregation by specific substances, such as alcohol, opioids, and stimulants, each possessing unique cardiovascular mechanisms and epidemiological patterns. Future research should investigate substance‐specific impacts on HF mortality to produce more clinically relevant results.

Third, the analysis did not account for individual‐level risk factors such as socioeconomic status, comorbidities, or access to care, which can substantially influence outcomes. Specifically, socioeconomic position and comorbidity burden are significant confounders that may independently influence both drug use disorders and HF mortality, and their exclusion from our analysis restricts the capacity to make causal or individual‐level inferences from the observed correlations.

Fourth, state‐level trend interpretation is constrained by methodological transitions in CDC WONDER reporting. State‐level analyses in this study were derived from two distinct CDC WONDER data sources with different methodological standards. Data from 1999 to 2020 (Supporting Information S1: Table 7) utilized bridged‐race population estimates from the traditional MCOD file, whereas data from 2021 to 2023 (Supporting Information S1: Table 11) were obtained from the expanded MCD file, which employs single‐race population estimates. This transition, implemented by the CDC following the discontinuation of bridged‐race estimates after the Vintage 2020 release [49], reflects the US Census Bureau's improvement to race and ethnicity data collection beginning with Census 2020 [50]. As these two sets of population estimates are based on incompatible racial classification systems and different underlying methodologies, data users are advised against making direct comparisons between statistics derived from bridged‐race and single‐race population estimates [50]. Consequently, while we present 2021−2023 state‐level estimates for descriptive completeness, these values should not be directly compared with the 1999−2020 estimates to infer temporal changes at the state level. This limitation parallels the urban−rural classification transition noted in our methods, where post‐2020 urbanization data were similarly non‐comparable due to the CDC's shift from a binary to a multi‐level urbanization framework. This same bridged‐race‐to‐single‐race transition is also reflected in Supporting Information S1: Table 1, where the number of deaths categorized by race/ethnicity does not fully sum to the overall total in any year, as deaths with Hispanic origin “Not Stated” are excluded from Hispanic‐origin–based categories per CDC WONDER reporting conventions; this discrepancy is more pronounced in 2021–2023 than in 1999–2020, consistent with differences in Hispanic‐origin reporting completeness between the two source data sets (see Supporting Information S1: Table 1, footnote). Note that this 2020/2021 methodological cutoff is distinct from the 2019/2020 cutoff used in the COVID‐19 pandemic stratification (Section 3.8), which instead reflects the pandemic's onset rather than a change in data source.

Fifth, geographic disparities may reflect variations in reporting standards, healthcare access, or substance use patterns across states and regions. Finally, while trends were stratified by demographic variables such as sex, age, race, and geography, the study did not explore potential confounding interactions between these variables, limiting causal interpretation.

## 6 Conclusion

This study demonstrates a substantial increase in HF and psychoactive substance abuse‐related mortality in the United States from 1999 to 2023, with the AAMR increasing over tenfold during this period. Although recent years have shown a slight decline, the overall burden remains substantial, especially among males, older adults, NH White populations, and those residing in nonmetropolitan and Midwestern regions. These findings underscore the growing public health challenge posed by the intersection of cardiovascular disease and substance use disorders. The geographic and demographic disparities highlight the need for targeted prevention strategies, improved access to addiction treatment, and integrated cardiovascular care, especially in high‐burden regions. Continued surveillance and deeper investigation into underlying causes are essential for effective policy and intervention planning.

## Author Contributions

All authors have equally contributed to the manuscript and have approved the final manuscript to be published.

## Funding

The authors have nothing to report.

## Ethics Statement

The authors have nothing to report. This study used publicly available, de‐identified, aggregate mortality data from the CDC WONDER database; no identifiable patient‐level data were accessed, and no direct patient contact occurred.

## Conflicts of Interest

The authors declare no conflicts of interest.

## Supporting information


Supporting File

## Data Availability

The data underlying this study are publicly available from the CDC WONDER Multiple Cause of Death database (https://wonder.cdc.gov/mcd.html). The specific data set extracted and analyzed for this study, along with the analytic code used to generate the reported estimates, is available from the corresponding author upon reasonable request.
