# Supplementary material for: Mortality Trend in Patients With Heart Failure and Psychoactive Substance Abuse in the United States—Pre‐ and Post‐COVID‐19 Pandemic Perspective (1999−2023)
Source: Clin Cardiol. 2026 Jul 27;49(8):e70425. doi: 10.1002/clc.70425 (PMC13405178; doi:10.1002/clc.70425)
Supplement: Supplementary file 1 — Supporting File [file CLC-49-e70425-s001.docx]

| **Supplemental Table 1, Number of mortalities due to Heart Failure with Psychoactive substance abuse stratified by overall, sex and race from 1999-2023** | | | | | | | | |
| --- | --- | --- | --- | --- | --- | --- | --- | --- |
| **Deaths** | | | | | | | | |
| **Year** | **Overall** | **Female** | **Male** | **Hispanics** | **NH Black** | **NH White** | **NH Asian and Others** | **Population** |
| **1999** | 2938 | 1053 | 1885 | 114 | 333 | 2441 | 42 | 180408769 |
| **2000** | 3977 | 1534 | 2443 | 127 | 383 | 3400 | 54 | 181984640 |
| **2001** | 3803 | 1439 | 2364 | 137 | 392 | 3203 | 60 | 184305128 |
| **2002** | 3978 | 1552 | 2426 | 111 | 358 | 3424 | 68 | 186208028 |
| **2003** | 10569 | 4140 | 6429 | 433 | 838 | 9171 | 105 | 188090429 |
| **2004** | 14582 | 5867 | 8715 | 413 | 1011 | 12930 | 191 | 190205384 |
| **2005** | 18395 | 7350 | 11045 | 517 | 1388 | 16230 | 228 | 192551384 |
| **2006** | 19645 | 7825 | 11820 | 610 | 1383 | 17346 | 273 | 195019359 |
| **2007** | 21067 | 8292 | 12775 | 622 | 1503 | 18597 | 309 | 197403777 |
| **2008** | 23956 | 9411 | 14545 | 614 | 1702 | 21240 | 351 | 199795090 |
| **2009** | 23549 | 9231 | 14318 | 623 | 1812 | 20738 | 330 | 202107016 |
| **2010** | 25995 | 10195 | 15800 | 712 | 1995 | 22879 | 354 | 203891983 |
| **2011** | 28501 | 11047 | 17454 | 710 | 2130 | 25251 | 361 | 206592936 |
| **2012** | 30973 | 12036 | 18937 | 848 | 2287 | 27362 | 387 | 208826037 |
| **2013** | 33945 | 12932 | 21013 | 972 | 2753 | 29649 | 506 | 211085314 |
| **2014** | 36156 | 13750 | 22406 | 1057 | 3035 | 31372 | 573 | 213809280 |
| **2015** | 41170 | 15718 | 25452 | 1295 | 3571 | 35525 | 626 | 216553817 |
| **2016** | 43880 | 16597 | 27283 | 1297 | 3977 | 37827 | 649 | 218641417 |
| **2017** | 47648 | 18030 | 29618 | 1476 | 4399 | 40903 | 741 | 221447331 |
| **2018** | 51390 | 19313 | 32077 | 1607 | 4730 | 44055 | 872 | 223311190 |
| **2019** | 54214 | 20501 | 33713 | 1723 | 5074 | 46360 | 932 | 224981167 |
| **2020** | 57340 | 21378 | 35962 | 1992 | 5933 | 48202 | 1097 | 226635013 |
| **2021** | 57730 | 21674 | 36056 | 2023 | 5877 | 48369 | 978 | 228238412 |
| **2022** | 58552 | 22151 | 36401 | 2029 | 5966 | 49064 | 954 | 229508599 |
| **2023** | 57786 | 21973 | 35813 | 2113 | 5958 | 48293 | 870 | 231529762 |
| **Total** | **771739** | **294989** | **476750** | **24175** | **68788** | **663831** | **11911** | **5163131262** |
| Note: NH White, NH Black or African American, Hispanic, and NH Asian/Other categories do not sum to the overall total in any year. Per CDC WONDER documentation, deaths with Hispanic origin "Not Stated" are included in overall counts but are not distributed among Hispanic-origin–based race/ethnicity categories. This discrepancy is present throughout 1999–2023 but is larger in 2021–2023 (483–552 deaths/year) than in 1999–2020 (approximately 8–150 deaths/year), consistent with differences in Hispanic-origin reporting completeness between the bridged-race (1999–2020) and single-race (2021–2023) CDC WONDER datasets. | | | | | | | | |

| **Supplemental Table 2, Number of mortalities and Percentage of Death due to Heart Failure with Psychoactive Substance Abuse stratified by Place of death from 1999-2023** | | |  |
| --- | --- | --- | --- |
|  |  |  |  |
| **Place of Death** | **Number of mortalities** | **Percentages** |  |
| Medical Facility | 302,810 | 39.24% |  |
| Decedent's home | 263,413 | 34.14% |  |
| Hospice facility | 51,352 | 6.65% |  |
| Nursing homes | 125,847 | 16.30% |  |
| Other | 28,269 | 3.67% |  |
| **Total** | **771739** | 100% |  |

| **Supplemental Table 3: Number of mortalities from 1999 to 2023 stratified by Age group** | | | |  |
| --- | --- | --- | --- | --- |
|  |  |  |  |  |
| **Deaths** | | | |  |
| **Year** | **Age 25-45 years** | **Age 46-65 years** | **Age 65+ years** |  |
| **1999** | 129 | 708 | 2101 |  |
| **2000** | 118 | 913 | 2946 |  |
| **2001** | 157 | 918 | 2728 |  |
| **2002** | 154 | 985 | 2839 |  |
| **2003** | 209 | 1780 | 8580 |  |
| **2004** | 241 | 2259 | 12082 |  |
| **2005** | 227 | 2856 | 15312 |  |
| **2006** | 248 | 3041 | 16356 |  |
| **2007** | 238 | 3239 | 17590 |  |
| **2008** | 275 | 3660 | 20021 |  |
| **2009** | 234 | 3617 | 19698 |  |
| **2010** | 228 | 4147 | 21620 |  |
| **2011** | 279 | 4540 | 23682 |  |
| **2012** | 282 | 4885 | 25806 |  |
| **2013** | 313 | 5423 | 28209 |  |
| **2014** | 317 | 5826 | 30013 |  |
| **2015** | 359 | 6521 | 34290 |  |
| **2016** | 450 | 7170 | 36260 |  |
| **2017** | 513 | 7869 | 39266 |  |
| **2018** | 633 | 8501 | 42256 |  |
| **2019** | 645 | 9092 | 44477 |  |
| **2020** | 863 | 10523 | 45954 |  |
| **2021** | 985 | 10962 | 45783 |  |
| **2022** | 1040 | 10604 | 46908 |  |
| **2023** | 1009 | 10280 | 46497 |  |
| **Total** | **10146** | **130319** | **631274** |  |

| **Supplemental Table 4: Age-Adjusted Mortality Rates from 1999 to 2023 stratified by Age group** | | | |  |
| --- | --- | --- | --- | --- |
|  |  |  |  |  |
| **Year** | **Age-Adjusted Rate (95% CI) Age 25-45 years** | **Age-Adjusted Rate (95% CI) Age 46-65 years** | **Age-Adjusted Rate (95% CI) Age 65+ years** |  |
| **1999** | 0.16 (0.13 - 0.19) | 1.21 (1.12 - 1.30) | 6.06 (5.81 - 6.32) |  |
| **2000** | 0.15 (0.13 - 0.18) | 1.47 (1.37 - 1.56) | 8.46 (8.15 - 8.76) |  |
| **2001** | 0.21 (0.18 - 0.24) | 1.43 (1.34 - 1.52) | 7.71 (7.42 - 7.99) |  |
| **2002** | 0.21 (0.18 - 0.24) | 1.45 (1.36 - 1.54) | 7.95 (7.66 - 8.24) |  |
| **2003** | 0.26 (0.23 - 0.30) | 2.56 (2.44 - 2.68) | 23.76 (23.25 - 24.26) |  |
| **2004** | 0.32 (0.28 - 0.36) | 3.09 (2.97 - 3.22) | 33.13 (32.54 - 33.72) |  |
| **2005** | 0.32 (0.28 - 0.36) | 3.81 (3.67 - 3.95) | 41.32 (40.66 - 41.97) |  |
| **2006** | 0.32 (0.28 - 0.36) | 3.91 (3.77 - 4.05) | 43.46 (42.80 - 44.13) |  |
| **2007** | 0.32 (0.28 - 0.36) | 4.00 (3.87 - 4.14) | 45.95 (45.27 - 46.63) |  |
| **2008** | 0.32 (0.28 - 0.36) | 4.40 (4.26 - 4.54) | 51.36 (50.65 - 52.08) |  |
| **2009** | 0.32 (0.28 - 0.36) | 4.24 (4.10 - 4.38) | 49.58 (48.89 - 50.27) |  |
| **2010** | 0.26 (0.23 - 0.30) | 4.76 (4.61 - 4.90) | 53.71 (52.99 - 54.43) |  |
| **2011** | 0.37 (0.33 - 0.42) | 5.08 (4.93 - 5.22) | 57.20 (56.47 - 57.93) |  |
| **2012** | 0.37 (0.33 - 0.42) | 5.41 (5.26 - 5.56) | 60.55 (59.81 - 61.30) |  |
| **2013** | 0.42 (0.37 - 0.46) | 5.89 (5.73 - 6.04) | 64.34 (63.58 - 65.10) |  |
| **2014** | 0.37 (0.33 - 0.41) | 6.24 (6.08 - 6.41) | 66.69 (65.93 - 67.46) |  |
| **2015** | 0.47 (0.42 - 0.52) | 6.92 (6.75 - 7.09) | 74.13 (73.34 - 74.92) |  |
| **2016** | 0.58 (0.53 - 0.64) | 7.55 (7.37 - 7.73) | 76.51 (75.71 - 77.31) |  |
| **2017** | 0.64 (0.58 - 0.69) | 8.20 (8.02 - 8.39) | 80.53 (79.72 - 81.33) |  |
| **2018** | 0.79 (0.73 - 0.85) | 8.86 (8.67 - 9.05) | 84.15 (83.34 - 84.95) |  |
| **2019** | 0.79 (0.73 - 0.85) | 9.49 (9.29 - 9.69) | 86.15 (85.34 - 86.95) |  |
| **2020** | 1.05 (0.98 - 1.13) | 11.08 (10.86 - 11.30) | 86.76 (85.96 - 87.56) |  |
| **2021** | 1.21 (1.13 - 1.28) | 11.44 (11.22 - 11.66) | 88.82 (87.99 - 89.64) |  |
| **2022** | 1.21 (1.14 - 1.28) | 11.22 (11.00 - 11.44) | 85.86 (85.07 - 86.64) |  |
| **2023** | 1.16 (1.09 - 1.24) | 10.95 (10.73 - 11.16) | 84.01 (83.24 - 84.77) |  |
| **Total** | **0.50 (0.45-0.55)** | **5.79 (5.63-5.94)** | **54.73 (54.06-55.39)** |  |

| **Supplemental Table 5: Overall and sex stratified Age-Adjusted Mortality Rates related to Heart Failure with Psychoactive substance abuse from 1999-2023** | | | |
| --- | --- | --- | --- |
| **Age-Adjusted Rate (95% Confidence Interval)** | | | |
| **Year** | **Men** | **Women** | **Overall** |
| **1999** | 2.56 (2.44 - 2.67) | 1.03 (0.97 - 1.09) | 1.68 (1.62 - 1.74) |
| **2000** | 3.30 (3.17 - 3.44) | 1.45 (1.38 - 1.52) | 2.23 (2.16 - 2.30) |
| **2001** | 3.07 (2.94 - 3.20) | 1.39 (1.32 - 1.46) | 2.09 (2.03 - 2.16) |
| **2002** | 3.12 (2.99 - 3.25) | 1.46 (1.38 - 1.53) | 2.15 (2.08 - 2.22) |
| **2003** | 8.46 (8.25 - 8.67) | 3.75 (3.63 - 3.86) | 5.64 (5.54 - 5.75) |
| **2004** | 11.36 (11.12 - 11.60) | 5.24 (5.11 - 5.38) | 7.68 (7.56 - 7.81) |
| **2005** | 14.14 (13.87 - 14.41) | 6.44 (6.30 - 6.59) | 9.53 (9.39 - 9.67) |
| **2006** | 14.81 (14.54 - 15.08) | 6.73 (6.58 - 6.88) | 9.98 (9.84 - 10.12) |
| **2007** | 15.66 (15.38 - 15.93) | 7.04 (6.89 - 7.19) | 10.50 (10.36 - 10.64) |
| **2008** | 17.41 (17.12 - 17.69) | 7.85 (7.69 - 8.01) | 11.70 (11.55 - 11.85) |
| **2009** | 16.72 (16.45 - 17.00) | 7.56 (7.41 - 7.72) | 11.29 (11.15 - 11.44) |
| **2010** | 18.09 (17.81 - 18.38) | 8.25 (8.09 - 8.41) | 12.25 (12.10 - 12.40) |
| **2011** | 19.30 (19.01 - 19.60) | 8.74 (8.58 - 8.91) | 13.09 (12.94 - 13.25) |
| **2012** | 20.31 (20.02 - 20.61) | 9.32 (9.15 - 9.49) | 13.86 (13.71 - 14.02) |
| **2013** | 21.77 (21.47 - 22.07) | 9.82 (9.65 - 9.99) | 14.79 (14.63 - 14.95) |
| **2014** | 22.56 (22.26 - 22.86) | 10.23 (10.05 - 10.40) | 15.35 (15.19 - 15.51) |
| **2015** | 24.94 (24.63 - 25.25) | 11.41 (11.22 - 11.59) | 17.08 (16.92 - 17.25) |
| **2016** | 25.98 (25.66 - 26.29) | 11.87 (11.68 - 12.05) | 17.81 (17.65 - 17.98) |
| **2017** | 27.36 (27.05 - 27.68) | 12.57 (12.38 - 12.76) | 18.85 (18.68 - 19.02) |
| **2018** | 28.88 (28.56 - 29.20) | 13.18 (13.00 - 13.37) | 19.85 (19.68 - 20.03) |
| **2019** | 29.48 (29.16 - 29.80) | 13.67 (13.48 - 13.86) | 20.46 (20.29 - 20.64) |
| **2020** | 30.60 (30.28 - 30.92) | 14.09 (13.89 - 14.28) | 21.25 (21.07 - 21.43) |
| **2021** | 31.05 (30.72 - 31.37) | 14.73 (14.53 - 14.93) | 21.84 (21.66 - 22.03) |
| **2022** | 30.29 (29.97 - 30.60) | 14.34 (14.15 - 14.54) | 21.19 (21.02 - 21.36) |
| **2023** | 29.16 (28.85 - 29.46) | 14.21 (14.02 - 14.40) | 20.71 (20.54 - 20.89) |
| **Total** | **18.82 (18.55 - 19.08)** | **8.65 (8.50 - 8.81)** | **12.91 (12.77 - 13.06)** |

| **Supplemental Table 6: Race stratified Age-Adjusted Mortality Rates related to Heart Failure with Psychoactive substance abuse from 1999-2023** | | | | |
| --- | --- | --- | --- | --- |
| **Age-Adjusted Rate (95% Confidence Interval)** | | | | |
| **Year** | **Hispanics** | **NH Black** | **NH White** | **NH Asian and Others** |
| **1999** | 1.17 (0.95 - 1.40) | 2.02 (1.80 - 2.25) | 1.65 (1.59 - 1.72) | 0.78 (0.55 - 1.07) |
| **2000** | 1.15 (0.94 - 1.36) | 2.31 (2.08 - 2.55) | 2.27 (2.19 - 2.34) | 1.03 (0.76 - 1.36) |
| **2001** | 1.27 (1.05 - 1.50) | 2.35 (2.12 - 2.59) | 2.14 (2.06 - 2.21) | 0.92 (0.69 - 1.20) |
| **2002** | 0.96 (0.77 - 1.15) | 2.05 (1.83 - 2.27) | 2.28 (2.20 - 2.35) | 1.03 (0.79 - 1.32) |
| **2003** | 4.01 (3.62 - 4.41) | 4.92 (4.58 - 5.26) | 5.92 (5.80 - 6.04) | 1.56 (1.25 - 1.87) |
| **2004** | 3.73 (3.36 - 4.11) | 5.86 (5.49 - 6.23) | 8.26 (8.11 - 8.40) | 2.94 (2.50 - 3.37) |
| **2005** | 4.45 (4.05 - 4.85) | 7.94 (7.52 - 8.37) | 10.22 (10.07 - 10.38) | 3.22 (2.78 - 3.65) |
| **2006** | 4.98 (4.57 - 5.39) | 7.64 (7.22 - 8.05) | 10.75 (10.59 - 10.91) | 3.69 (3.23 - 4.14) |
| **2007** | 4.85 (4.46 - 5.25) | 8.21 (7.78 - 8.63) | 11.37 (11.21 - 11.53) | 4.00 (3.54 - 4.46) |
| **2008** | 4.59 (4.21 - 4.97) | 9.09 (8.65 - 9.54) | 12.80 (12.62 - 12.97) | 4.37 (3.90 - 4.84) |
| **2009** | 4.37 (4.01 - 4.72) | 9.46 (9.01 - 9.91) | 12.34 (12.17 - 12.51) | 3.91 (3.47 - 4.35) |
| **2010** | 4.83 (4.46 - 5.19) | 10.15 (9.70 - 10.61) | 13.40 (13.23 - 13.58) | 4.14 (3.70 - 4.59) |
| **2011** | 4.46 (4.12 - 4.80) | 10.41 (9.96 - 10.87) | 14.54 (14.36 - 14.72) | 3.87 (3.46 - 4.28) |
| **2012** | 5.06 (4.71 - 5.42) | 10.78 (10.33 - 11.24) | 15.42 (15.24 - 15.61) | 3.77 (3.39 - 4.16) |
| **2013** | 5.52 (5.16 - 5.87) | 12.42 (11.94 - 12.90) | 16.41 (16.22 - 16.60) | 4.67 (4.25 - 5.08) |
| **2014** | 5.59 (5.25 - 5.94) | 13.40 (12.91 - 13.89) | 17.08 (16.89 - 17.27) | 5.04 (4.62 - 5.47) |
| **2015** | 6.48 (6.12 - 6.84) | 15.32 (14.80 - 15.84) | 19.00 (18.80 - 19.20) | 5.09 (4.68 - 5.49) |
| **2016** | 6.08 (5.74 - 6.42) | 16.33 (15.81 - 16.85) | 19.90 (19.69 - 20.10) | 5.03 (4.63 - 5.42) |
| **2017** | 6.54 (6.20 - 6.88) | 17.45 (16.92 - 17.98) | 21.13 (20.92 - 21.33) | 5.46 (5.06 - 5.85) |
| **2018** | 6.92 (6.57 - 7.27) | 18.32 (17.78 - 18.86) | 22.33 (22.12 - 22.54) | 6.04 (5.63 - 6.45) |
| **2019** | 7.01 (6.67 - 7.35) | 19.03 (18.49 - 19.57) | 23.07 (22.86 - 23.29) | 6.19 (5.78 - 6.59) |
| **2020** | 7.60 (7.25 - 7.94) | 21.45 (20.89 - 22.01) | 23.79 (23.58 - 24.01) | 6.92 (6.51 - 7.34) |
| **2021** | 7.58 (7.23 - 7.92) | 21.62 (21.05 - 22.19) | 24.80 (24.57 - 25.02) | 6.47 (6.06 - 6.88) |
| **2022** | 7.27 (6.94 - 7.59) | 21.56 (21.00 - 22.13) | 24.10 (23.88 - 24.32) | 6.05 (5.66 - 6.44) |
| **2023** | 7.24 (6.92 - 7.56) | 21.05 (20.50 - 21.60) | 23.74 (23.52 - 23.96) | 5.24 (4.88 - 5.59) |
| **Total** | **4.95 (4.61 - 5.28)** | **11.65 (11.21 - 12.09)** | **14.35 (14.18 - 14.52)** | **4.06 (3.67 - 4.45)** |

| **Supplemental Table 7: Number of mortalities and Age-Adjusted Mortality Rates from 1999 to 2020 stratified by States** | | |
| --- | --- | --- |
| **State** | **Deaths** | **Age-Adjusted Rate (95% Confidence Interval)** |
| Alabama | 3546 | 4.64 (4.48 - 4.79) |
| Alaska | 937 | 14.97 (13.96 - 15.98) |
| Arizona | 9818 | 9.82 (9.63 - 10.02) |
| Arkansas | 7295 | 15.25 (14.90 - 15.61) |
| California | 11936 | 2.28 (2.24 - 2.32) |
| Colorado | 9770 | 14.79 (14.49 - 15.08) |
| Connecticut | 6636 | 10.67 (10.41 - 10.93) |
| Delaware | 2074 | 14.08 (13.47 - 14.69) |
| District of Columbia | 532 | 6.23 (5.70 - 6.76) |
| Florida | 31023 | 8.20 (8.10 - 8.29) |
| Georgia | 11072 | 8.87 (8.70 - 9.04) |
| Hawaii | 2273 | 9.90 (9.49 - 10.31) |
| Idaho | 6289 | 28.13 (27.43 - 28.83) |
| Illinois | 18409 | 9.51 (9.37 - 9.64) |
| Indiana | 17279 | 17.36 (17.10 - 17.62) |
| Iowa | 8266 | 15.07 (14.74 - 15.40) |
| Kansas | 8517 | 18.63 (18.23 - 19.03) |
| Kentucky | 12940 | 19.31 (18.98 - 19.65) |
| Louisiana | 8625 | 12.87 (12.60 - 13.15) |
| Maine | 3951 | 15.94 (15.45 - 16.44) |
| Maryland | 9401 | 11.12 (10.89 - 11.35) |
| Massachusetts | 5575 | 5.01 (4.87 - 5.14) |
| Michigan | 31708 | 19.73 (19.52 - 19.95) |
| Minnesota | 13044 | 15.59 (15.32 - 15.85) |
| Mississippi | 2580 | 5.75 (5.52 - 5.97) |
| Missouri | 13765 | 14.04 (13.80 - 14.28) |
| Montana | 4625 | 27.16 (26.38 - 27.95) |
| Nebraska | 7068 | 23.67 (23.11 - 24.23) |
| Nevada | 3413 | 9.71 (9.37 - 10.04) |
| New Hampshire | 4375 | 20.63 (20.01 - 21.24) |
| New Jersey | 16149 | 11.29 (11.11 - 11.46) |
| New Mexico | 4265 | 13.91 (13.49 - 14.32) |
| New York | 33968 | 10.75 (10.64 - 10.87) |
| North Carolina | 16700 | 11.63 (11.46 - 11.81) |
| North Dakota | 3495 | 29.14 (28.16 - 30.12) |
| Ohio | 31844 | 16.55 (16.36 - 16.73) |
| Oklahoma | 12203 | 21.04 (20.66 - 21.41) |
| Oregon | 22688 | 35.92 (35.45 - 36.39) |
| Pennsylvania | 26203 | 11.12 (10.99 - 11.26) |
| Rhode Island | 3162 | 16.48 (15.90 - 17.06) |
| South Carolina | 12291 | 16.98 (16.68 - 17.28) |
| South Dakota | 3307 | 23.16 (22.36 - 23.95) |
| Tennessee | 12388 | 12.59 (12.37 - 12.81) |
| Texas | 57152 | 18.59 (18.44 - 18.75) |
| Utah | 4213 | 14.32 (13.89 - 14.76) |
| Vermont | 3197 | 29.96 (28.92 - 31.01) |
| Virginia | 7475 | 6.46 (6.31 - 6.60) |
| Washington | 26126 | 26.52 (26.19 - 26.84) |
| West Virginia | 2390 | 7.05 (6.76 - 7.33) |
| Wisconsin | 19493 | 20.80 (20.50 - 21.09) |
| Wyoming | 2220 | 27.38 (26.23 - 28.52) |

| **Supplemental Table 8: Age-Adjusted Mortality Rates from 1999 to 2023 stratified by Census Region** | | | | |
| --- | --- | --- | --- | --- |
| **Year** | **Age-Adjusted Rate - Northeast** | **Age-Adjusted Rate - Midwest** | **Age-Adjusted Rate - South** | **Age-Adjusted Rate - West** |
| **1999** | 0.87 (0.78 - 0.97) | 1.59 (1.47 - 1.71) | 1.73 (1.63 - 1.83) | 2.43 (2.27 - 2.59) |
| **2000** | 1.22 (1.10 - 1.33) | 2.08 (1.95 - 2.22) | 2.45 (2.33 - 2.57) | 2.96 (2.78 - 3.14) |
| **2001** | 1.18 (1.07 - 1.29) | 2.09 (1.96 - 2.23) | 2.24 (2.12 - 2.36) | 2.77 (2.60 - 2.94) |
| **2002** | 1.21 (1.10 - 1.31) | 2.15 (2.01 - 2.29) | 2.23 (2.12 - 2.34) | 2.91 (2.74 - 3.09) |
| **2003** | 4.90 (4.68 - 5.12) | 4.40 (4.21 - 4.60) | 6.56 (6.37 - 6.76) | 6.14 (5.89 - 6.39) |
| **2004** | 7.92 (7.64 - 8.20) | 7.37 (7.12 - 7.62) | 6.92 (6.72 - 7.12) | 9.01 (8.71 - 9.31) |
| **2005** | 8.99 (8.69 - 9.28) | 9.43 (9.15 - 9.72) | 10.08 (9.84 - 10.31) | 9.07 (8.77 - 9.37) |
| **2006** | 12.18 (11.84 - 12.52) | 9.29 (9.01 - 9.57) | 9.34 (9.12 - 9.57) | 9.57 (9.27 - 9.87) |
| **2007** | 11.90 (11.56 - 12.23) | 12.02 (11.70 - 12.34) | 9.26 (9.04 - 9.49) | 9.48 (9.18 - 9.78) |
| **2008** | 12.41 (12.07 - 12.75) | 15.99 (15.63 - 16.36) | 9.59 (9.37 - 9.82) | 9.83 (9.53 - 10.13) |
| **2009** | 11.82 (11.49 - 12.15) | 15.65 (15.30 - 16.01) | 9.36 (9.14 - 9.58) | 9.29 (9.00 - 9.58) |
| **2010** | 11.95 (11.61 - 12.28) | 16.89 (16.52 - 17.26) | 10.61 (10.38 - 10.84) | 10.36 (10.06 - 10.66) |
| **2011** | 11.88 (11.55 - 12.21) | 19.96 (19.56 - 20.36) | 10.74 (10.51 - 10.97) | 10.81 (10.51 - 11.11) |
| **2012** | 12.46 (12.13 - 12.80) | 20.41 (20.01 - 20.81) | 12.01 (11.77 - 12.24) | 11.32 (11.02 - 11.63) |
| **2013** | 13.50 (13.15 - 13.84) | 21.45 (21.04 - 21.85) | 12.88 (12.63 - 13.12) | 12.22 (11.91 - 12.53) |
| **2014** | 13.17 (12.83 - 13.51) | 22.42 (22.01 - 22.83) | 14.30 (14.04 - 14.55) | 11.89 (11.59 - 12.19) |
| **2015** | 15.07 (14.70 - 15.43) | 23.43 (23.01 - 23.84) | 16.46 (16.19 - 16.72) | 13.43 (13.11 - 13.75) |
| **2016** | 15.08 (14.72 - 15.44) | 24.53 (24.11 - 24.96) | 17.24 (16.97 - 17.51) | 14.31 (13.98 - 14.63) |
| **2017** | 15.56 (15.20 - 15.92) | 26.24 (25.80 - 26.67) | 18.47 (18.20 - 18.75) | 14.95 (14.63 - 15.28) |
| **2018** | 16.18 (15.82 - 16.55) | 26.93 (26.50 - 27.37) | 20.30 (20.01 - 20.58) | 15.24 (14.92 - 15.57) |
| **2019** | 16.22 (15.86 - 16.57) | 27.51 (27.07 - 27.94) | 21.04 (20.75 - 21.33) | 16.09 (15.76 - 16.42) |
| **2020** | 16.46 (16.10 - 16.82) | 29.82 (29.37 - 30.27) | 21.36 (21.07 - 21.64) | 16.70 (16.37 - 17.03) |
| **2021** | 16.21 (15.85 - 16.57) | 30.59 (30.13 - 31.06) | 22.03 (21.73 - 22.32) | 17.75 (17.41 - 18.10) |
| **2022** | 15.65 (15.31 - 16.00) | 29.43 (28.98 - 29.87) | 21.37 (21.08 - 21.65) | 17.64 (17.31 - 17.98) |
| **2023** | 15.24 (14.89 - 15.58) | 28.88 (28.44 - 29.32) | 21.04 (20.76 - 21.32) | 17.06 (16.73 - 17.39) |
| **Total** | **11.17 (10.87 - 11.47)** | **17.22 (16.88 - 17.56)** | **12.38 (12.16 - 12.61)** | **10.93 (10.64 - 11.22)** |

| **Supplemental Table 9: Age-Adjusted Mortality Rates from 1999 to 2020 stratified by Urbanization** | | |
| --- | --- | --- |
| **Year** | **Urban Age-Adjusted Rate** | **Rural Age-Adjusted Rate** |
| **1999** | 1.51 (1.44 - 1.57) | 2.32 (2.16 - 2.49) |
| **2000** | 2.00 (1.93 - 2.07) | 3.26 (3.07 - 3.46) |
| **2001** | 1.88 (1.81 - 1.95) | 3.11 (2.92 - 3.30) |
| **2002** | 1.94 (1.87 - 2.01) | 3.17 (2.98 - 3.36) |
| **2003** | 5.02 (4.91 - 5.14) | 8.38 (8.08 - 8.69) |
| **2004** | 6.91 (6.78 - 7.04) | 11.13 (10.78 - 11.48) |
| **2005** | 8.70 (8.56 - 8.85) | 13.14 (12.77 - 13.52) |
| **2006** | 9.21 (9.06 - 9.36) | 13.39 (13.01 - 13.77) |
| **2007** | 9.67 (9.52 - 9.82) | 14.27 (13.88 - 14.66) |
| **2008** | 10.67 (10.51 - 10.82) | 16.46 (16.04 - 16.87) |
| **2009** | 10.37 (10.21 - 10.52) | 15.61 (15.21 - 16.01) |
| **2010** | 11.05 (10.89 - 11.21) | 17.86 (17.43 - 18.29) |
| **2011** | 11.69 (11.53 - 11.85) | 19.58 (19.13 - 20.03) |
| **2012** | 12.36 (12.20 - 12.52) | 20.89 (20.44 - 21.35) |
| **2013** | 13.23 (13.06 - 13.39) | 22.24 (21.77 - 22.71) |
| **2014** | 13.77 (13.60 - 13.94) | 23.14 (22.67 - 23.61) |
| **2015** | 15.21 (15.04 - 15.39) | 26.27 (25.77 - 26.77) |
| **2016** | 15.96 (15.78 - 16.14) | 26.94 (26.43 - 27.45) |
| **2017** | 16.89 (16.71 - 17.07) | 28.67 (28.16 - 29.19) |
| **2018** | 17.79 (17.61 - 17.97) | 30.43 (29.90 - 30.96) |
| **2019** | 18.31 (18.13 - 18.49) | 31.34 (30.81 - 31.88) |
| **2020** | 19.00 (18.81 - 19.18) | 32.77 (32.23 - 33.32) |
| **Total** | **11.29 (11.25 - 11.32)** | **18.20 (18.11 - 18.29)** |

| **Supplemental Table 10: Annual percent change (APC) of Heart Failure related Age-Adjusted Mortality Rates per 100,000, with Psychoactive substance abuse, 1999 to 2023** | | | | |  |
| --- | --- | --- | --- | --- | --- |
|  |  |  |  |  |  |
| **Category** | **Subgroup** | **Years** | **APC (95% Confidence Interval)** | **P-value** |  |
| **Overall** | All | 1999–2005 | 38.53* (29.12 to 55.86) | 0.002 |  |
|  |  | 2005–2020 | 5.69* (5.03 to 14.82) | < 0.001 |  |
|  |  | 2020–2023 | -1.53 (-11.02 to 4.41) | 0.534 |  |
|  |  |  |  |  |  |
| **Sex** | Female | 1999–2005 | 38.26* (29.58 to 50.04) | 0.001 |  |
|  |  | 2005–2020 | 5.58* (5.04 to 14.03) | < 0.001 |  |
|  |  | 2020–2023 | -0.51 (-8.36 to 4.55) | 0.855 |  |
|  |  |  |  |  |  |
|  | Male | 1999–2005 | 38.37* (29.32 to 53.22) | < 0.001 |  |
|  |  | 2005–2020 | 5.45* (4.81 to 7.15) | < 0.001 |  |
|  |  | 2020–2023 | -2.30 (-11.65 to 2.56) | 0.295 |  |
|  |  |  |  |  |  |
| **Census** | Northeast | 1999–2005 | 59.55* (43.55 to 76.67) | < 0.001 |  |
|  |  | 2005–2023 | 2.37* (1.63 to 3.21) | < 0.001 |  |
|  |  |  |  |  |  |
|  | Midwest | 1999–2008 | 29.33* (23.76 to 38.19) | 0.007 |  |
|  |  | 2008–2021 | 5.07* (4.33 to 28.85) | 0.006 |  |
|  |  | 2021–2023 | -4.06 (-9.38 to 4.45) | 0.437 |  |
|  |  |  |  |  |  |
|  | South | 1999–2005 | 35.77* (27.98 to 49.50) | 0.005 |  |
|  |  | 2005–2009 | -0.83 (-7.92 to 6.08) | 0.841 |  |
|  |  | 2009–2019 | 8.75* (7.49 to 15.91) | < 0.001 |  |
|  |  | 2019–2023 | -0.69 (-6.83 to 3.06) | 0.686 |  |
|  |  |  |  |  |  |
|  | West | 1999–2001 | -0.68 (-16.06 to 32.03) | 0.949 |  |
|  |  | 2001–2004 | 48.32* (3.68 to 61.25) | 0.003 |  |
|  |  | 2004–2023 | 4.20* (3.65 to 4.81) | 0.010 |  |
|  |  |  |  |  |  |
| **Race** | Hispanic | 1999–2004 | 36.43* (21.12 to 93.01) | < 0.001 |  |
|  |  | 2004–2023 | 3.42* (2.53 to 4.47) | < 0.001 |  |
|  |  |  |  |  |  |
|  | NH Black | 1999–2005 | 27.23* (20.32 to 41.15) | 0.004 |  |
|  |  | 2005–2020 | 7.57* (6.90 to 8.96) | < 0.001 |  |
|  |  | 2020–2023 | 0.18 (-8.15 to 4.53) | 0.860 |  |
|  |  |  |  |  |  |
|  | NH White | 1999–2005 | 38.89* (30.55 to 49.59) | < 0.001 |  |
|  |  | 2005–2020 | 5.99* (5.40 to 7.48) | < 0.001 |  |
|  |  | 2020–2023 | -1.11 (-9.51 to 3.49) | 0.607 |  |
|  |  |  |  |  |  |
|  | NH Asian and Others | 1999–2005 | 32.30* (22.62 to 53.50) | 0.002 |  |
|  |  | 2005–2021 | 4.49* (3.76 to 5.80) | < 0.001 |  |
|  |  | 2021–2023 | -11.19* (-18.27 to -1.98) | 0.013 |  |
|  |  |  |  |  |  |
| **Urbanization** | Urban | 1999–2005 | 37.50* (29.43 to 48.82) | < 0.001 |  |
|  |  | 2005–2020 | 5.51* (4.84 to 6.34) | < 0.001 |  |
|  |  |  |  |  |  |
|  | Rural | 1999–2001 | 5.79 (-14.12 to 49.76) | 0.546 |  |
|  |  | 2001–2004 | 61.11* (5.20 to 79.54) | 0.002 |  |
|  |  | 2004–2020 | 6.72* (5.43 to 8.42) | 0.011 |  |
|  |  |  |  |  |  |
| **Age group** | Age 25-45 years | 1999–2004 | 16.41* (9.70 to 41.51) | < 0.001 |  |
|  |  | 2004–2014 | 2.80 (-2.24 to 4.53) | 0.131 |  |
|  |  | 2014–2021 | 17.41* (15.12 to 23.87) | < 0.001 |  |
|  |  | 2021–2023 | -1.59 (-8.69 to 7.28) | 0.703 |  |
|  |  |  |  |  |  |
|  | Age 46-65 years | 1999–2005 | 23.17* (18.77 to 31.07) | 0.002 |  |
|  |  | 2005–2011 | 4.87 (-0.12 to 7.28) | 0.056 |  |
|  |  | 2011–2021 | 8.88* (8.25 to 12.33) | < 0.001 |  |
|  |  | 2021–2023 | -2.68 (-7.60 to 2.89) | 0.292 |  |
|  |  |  |  |  |  |
|  | Age 65+ years | 1999–2001 | 0.64 (-18.55 to 45.48) | 0.934 |  |
|  |  | 2001–2004 | 75.80* (6.60 to 95.56) | < 0.001 |  |
|  |  | 2004–2018 | 6.04* (5.30 to 7.09) | 0.008 |  |
|  |  | 2018–2023 | -0.08 (-3.35 to 1.97) | 0.953 |  |

| **Supplemental Table 11: Number of mortalities and Age-Adjusted Mortality Rates from 2021 to 2023 stratified by States** | | |
| --- | --- | --- |
| **State** | **Deaths** | **Age-Adjusted Rate (95% Confidence Interval)** |
| Alabama | 1859 | 14.62 (13.93-15.3) |
| Alaska | 512 | 37.74 (34.31-41.17) |
| Arizona | 3659 | 19.18 (18.55-19.82) |
| Arkansas | 1936 | 25.44 (24.29-26.6) |
| California | 3345 | 3.76 (3.63-3.89) |
| Colorado | 3274 | 26.33 (25.41-27.25) |
| Connecticut | 969 | 9.89 (9.26-10.52) |
| Delaware | 800 | 28.5 (26.48-30.52) |
| District of Columbia | 125 | 9.58 (7.88-11.28) |
| Florida | 9125 | 13.35 (13.07-13.63) |
| Georgia | 3657 | 15.28 (14.78-15.79) |
| Hawaii | 667 | 16.71 (15.4-18.03) |
| Idaho | 1641 | 36.75 (34.94-38.56) |
| Illinois | 5465 | 17.49 (17.02-17.96) |
| Indiana | 5682 | 34.78 (33.87-35.7) |
| Iowa | 2796 | 33.7 (32.43-34.97) |
| Kansas | 2251 | 31.52 (30.2-32.84) |
| Kentucky | 5010 | 45.46 (44.18-46.74) |
| Louisiana | 2927 | 26.91 (25.92-27.91) |
| Maine | 1462 | 34.59 (32.78-36.4) |
| Maryland | 2523 | 16.71 (16.05-17.36) |
| Massachusetts | 2798 | 15.5 (14.92-16.08) |
| Michigan | 8133 | 31.32 (30.62-32.01) |
| Minnesota | 5720 | 40.44 (39.38-41.5) |
| Mississippi | 1281 | 17.75 (16.76-18.75) |
| Missouri | 4702 | 29.94 (29.07-30.81) |
| Montana | 948 | 32 (29.92-34.08) |
| Nebraska | 1543 | 32.84 (31.18-34.51) |
| Nevada | 1866 | 26.07 (24.86-27.27) |
| New Hampshire | 1219 | 31.36 (29.57-33.15) |
| New Jersey | 2992 | 12.71 (12.25-13.17) |
| New Mexico | 997 | 18.26 (17.11-19.42) |
| New York | 6394 | 12.29 (11.99-12.6) |
| North Carolina | 7451 | 28.45 (27.79-29.11) |
| North Dakota | 641 | 34.39 (31.67-37.1) |
| Ohio | 8716 | 28.64 (28.03-29.25) |
| Oklahoma | 2465 | 26.58 (25.51-27.65) |
| Oregon | 5874 | 53.64 (52.25-55.03) |
| Pennsylvania | 6664 | 18.34 (17.89-18.78) |
| Rhode Island | 728 | 24.52 (22.71-26.32) |
| South Carolina | 3328 | 24.42 (23.57-25.27) |
| South Dakota | 828 | 36.82 (34.25-39.38) |
| Tennessee | 5261 | 30.67 (29.83-31.52) |
| Texas | 12996 | 22.59 (22.19-22.98) |
| Utah | 1229 | 21.68 (20.45-22.91) |
| Vermont | 741 | 39.82 (36.9-42.73) |
| Virginia | 4612 | 22.02 (21.38-22.67) |
| Washington | 7341 | 40.99 (40.04-41.94) |
| West Virginia | 1461 | 28.35 (26.86-29.83) |
| Wisconsin | 4866 | 31.82 (30.91-32.72) |
| Wyoming | 588 | 40.71 (37.35-44.07) |


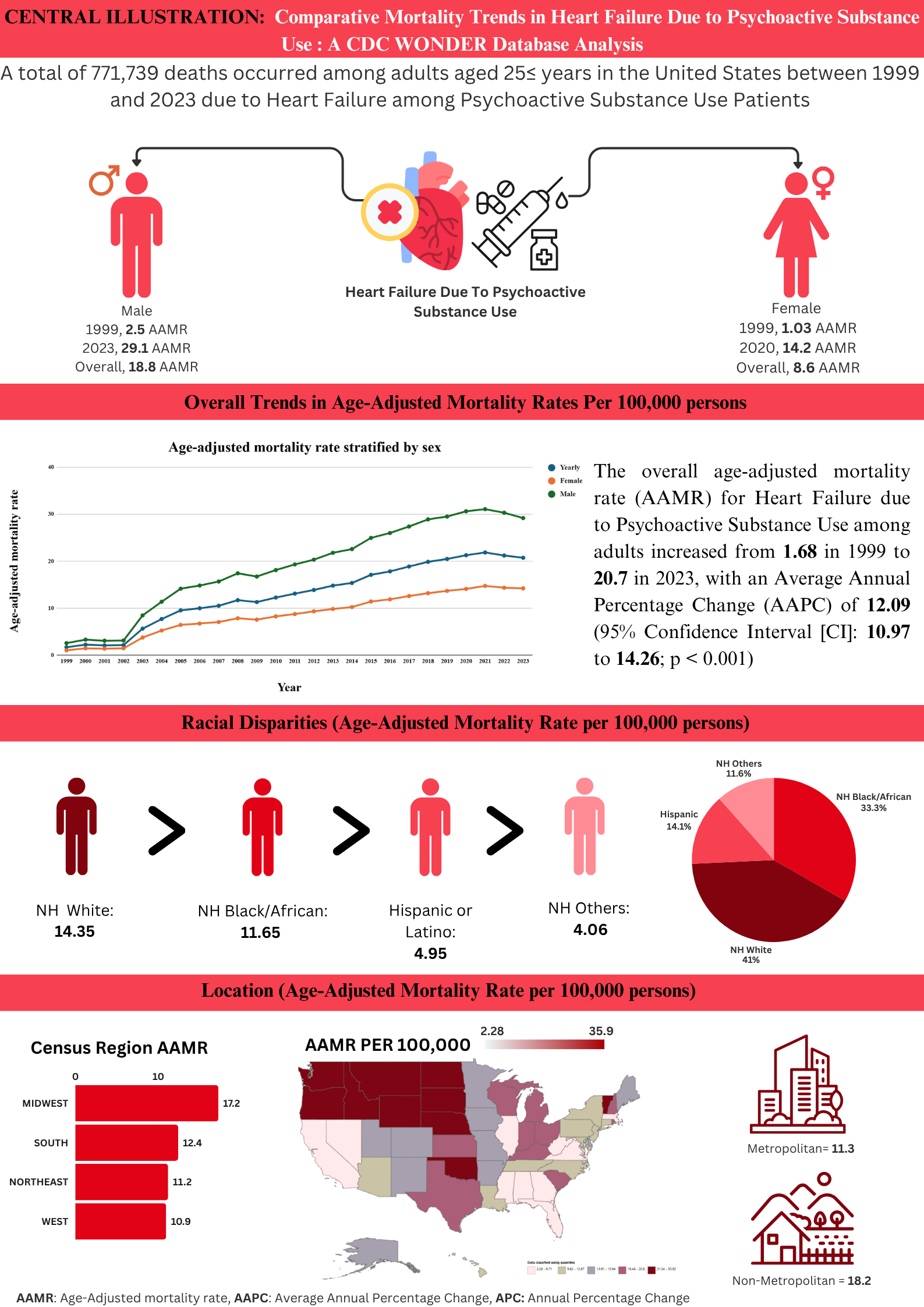


**Figure 1: Central Image for patients with Heart failure and Psychoactive substance abuse.**


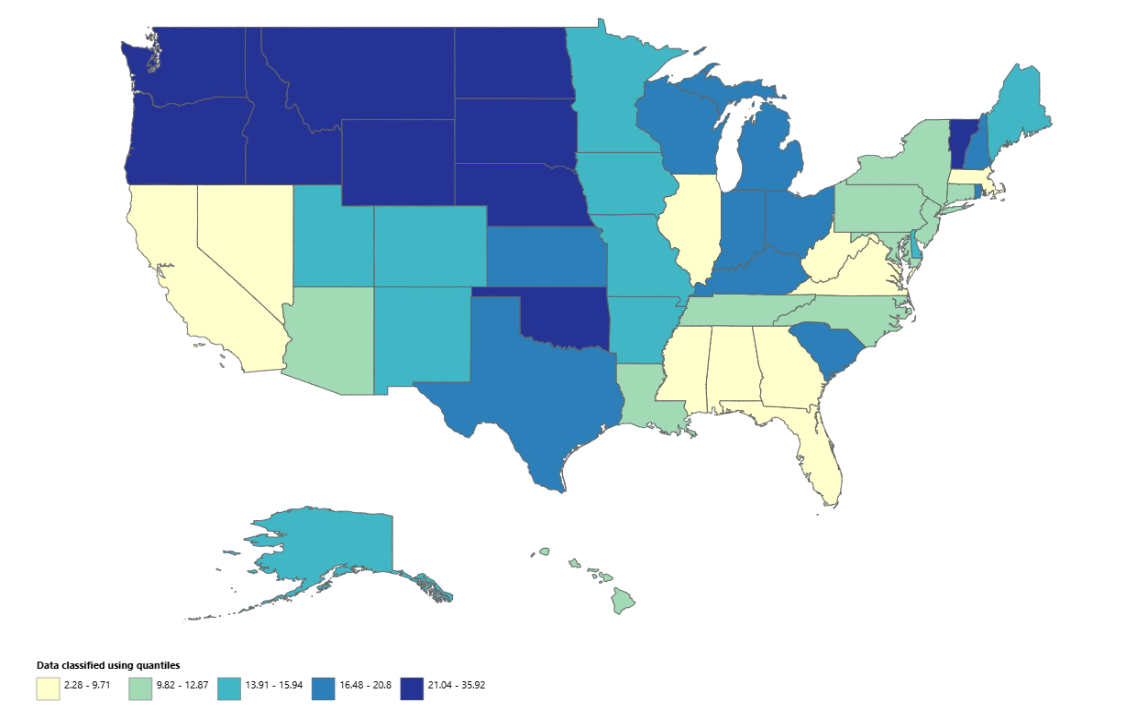


**Figure 2: A map displays the differences in age-adjusted mortality rates among U.S. states for patients with Heat failure and Psychoactive substance abuse.**

**Figure 3: Temporal trends of Age-Adjusted Mortality Rate in patients with Heart Failure and Psychoactive substance abuse stratified by sex**

**Figure 4: Temporal trends of Age-Adjusted Mortality Rate in patients with Heart Failure and Psychoactive substance abuse stratified by race**

**Figure 5: Temporal trends of Age-Adjusted Mortality Rate in patients with Heart Failure and Psychoactive substance abuse stratified by census region**

**Figure 6: Temporal trends of Age-Adjusted Mortality Rate in patients with Heart Failure and Psychoactive substance abuse stratified by urbanization**

**Figure 7: Temporal trends of Age-Adjusted Mortality Rate in patients with Heart Failure and Psychoactive substance abuse stratified by age**


 **Figure 8: Race and Region-wise analysis of AAMR per 100,000 in Heart Failure and Psychoactive Substance Abuse related Mortality in Adults from 1999-2019.


Figure 9: Race and Region-wise analysis of AAMR per 100,000 in Heart Failure and Psychoactive Substance Abuse related Mortality in Adults from 2020-2023.**


**Figure 10: Percentage of Place of Death in Heart Failure and Psychoactive Substance Abuse related Mortality in Adults from 1999-2023.**
